# Predicting frictional aging from bulk relaxation measurements

**DOI:** 10.1038/s41467-023-39350-3

**Published:** 2023-06-17

**Authors:** Kasra Farain, Daniel Bonn

**Affiliations:** Van der Waals–Zeeman Institute, Institute of Physics, University of Amsterdam, Science Park 904, 1098 XH Amsterdam, Netherlands

**Keywords:** Statistical physics, thermodynamics and nonlinear dynamics, Materials science

## Abstract

The coefficient of static friction between solids normally increases with the time they have remained in static contact before the measurement. This phenomenon, known as frictional aging, is at the origin of the difference between static and dynamic friction coefficients but has remained difficult to understand. It is usually attributed to a slow expansion of the area of atomic contact as the interface changes under pressure. This is however challenging to quantify as surfaces have roughness at all length scales. In addition, friction is not always proportional to the contact area. Here we show that the normalized stress relaxation of the surface asperities during frictional contact with a hard substrate is the same as that of the bulk material, regardless of the asperities’ size or degree of compression. This result enables us to predict the frictional aging of rough interfaces based on the bulk material properties of two typical polymers: polypropylene and polytetrafluoroethylene.

## Abstract (web summary)

Increase of friction between two solid surfaces in stationary contact over time, known as frictional aging, has been widely observed. Farain and Bonn show that, regardless of surface roughness or degree of compression, the normalized stress relaxation of microcontacts is the same as that of bulk material.

## Introduction

The contact between common solids involves time-dependent localized surface deformations. When two rough surfaces are pressed together, the mechanical interaction between them occurs through a force-bearing ensemble of microcontact^1–8^. Depending on the surface roughness and material characteristics, the local stress on the contact area can easily reach the yield stress of the material. As a result, some parts of the contact area will exhibit plastic flow while other less-strained parts will contribute to supporting the total normal load elastically^4,9,10^. This highly complex elastoplastic problem is at the heart of a fundamental understanding of many important surface and interface phenomena, from friction and adhesion to sealing and interfacial stiffness.

For friction, Amontons’ law states that the frictional force between two bodies is proportional to the normal load acting between them and is independent of the macroscopic area of contact. These are usually understood as a consequence of the surface roughness: the area of atomic contact between the two bodies A is only a small fraction of the macroscopic contact area and can be proportional to the normal load F in some simple models. Assuming that the overall shear strength of an interface is constant during sliding, one recovers Amontons’ law^1,4,11^. For over a century, various experimental techniques from electrical conductance measurements to optical and recently fluorescence observations have investigated the relation between A and F under a broad range of conditions^3,4,11,12^. However, a full analytical or computational understanding of this problem, even when reduced to an equilibrium problem, is still lacking. Early work first focused on plastic changes in contact area^11^. Later, purely elastic deformations of independent surface asperities were considered^13–15^. However, contact area measurements show that plastic deformations cannot be neglected and that the elastic coupling between the asperities is also important^9,16^. Recently, a contact mechanics theory was developed by Persson that can take into account plastic deformations by supposing that wherever the yield criterion (e.g., the von Mises yield condition) of the softer solid is satisfied, (instant) plastic changes occur^7,17,18^. Finite-element calculations also use such a fixed yield criterion^9,16,19–21^. This however cannot account for experimentally observed slow aging dynamics of static frictional interfaces that leads to the difference between static and dynamic friction^3,4,22,23^. In the simplest scenario and under a constant load, the contact area and frictional strength grow simultaneously with the logarithm of time^3,4^. Consequently, frictional aging is usually associated with contact area creep^3,23,24^. However, recent work shows that friction force and area may even evolve in different directions^4,8^. Settling these issues is all the more important as the slow evolution of frictional interfaces constitutes the basis of transient sliding dynamics at low velocities (e.g., rate and state friction transients), emerging frictional instabilities, and slip-stick phenomena^1,2,25^.

In the current work, we investigate the evolution of friction due to stress relaxation of surface irregularities in polymers in contact with a rigid surface. We first study the stress relaxation of polymers after macroscale bulk and/or microscale surface deformations. We find a simple and generic expression for the stress relaxation dynamics without any significant signature of the surface structure. We then show that the same time dependence also emerges from friction measurements, indicating that stress relaxation determines frictional aging.

## Results

### Stress relaxation

**Fig. 1:**
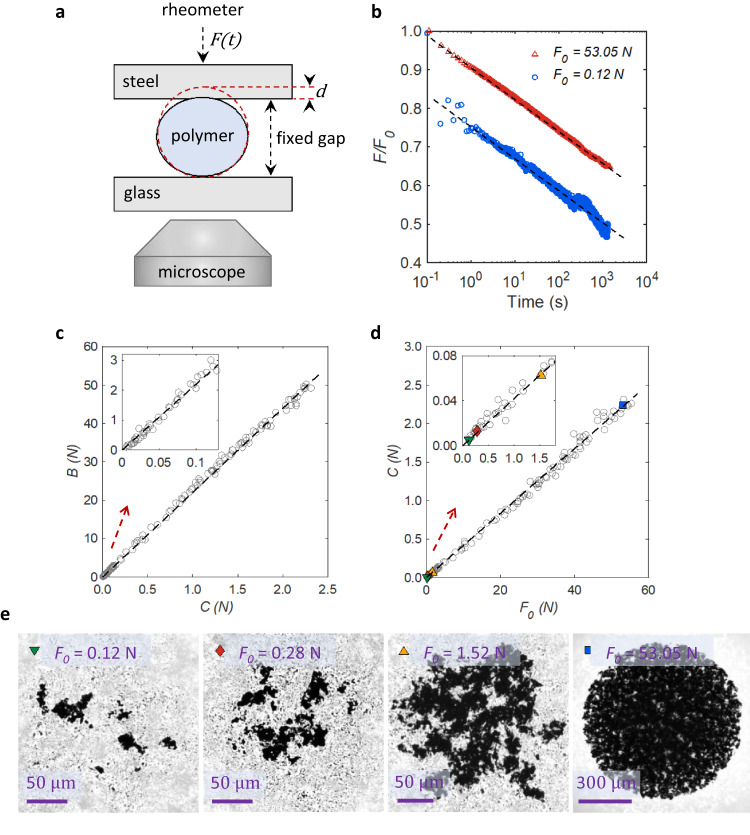
Stress relaxation of the polymer spheres after bulk and/or surface deformations. **a** Schematic of the setup for deformation experiments. A polypropylene or polytetrafluoroethylene sphere is squeezed between a steel plate connected to a rheometer and a glass slide installed on a microscope via a very stiff frame. **b** Relaxation of the applied force *F* needed to maintain a constant strain as a function of time for two values of the initial force ${F}_{0}$; 0.12 N (blue circles) and 53.05 N (red triangles). Dashed lines indicate logarithmic relaxation, Eq. 1. **c** Relation between constants *C* and *B* as obtained from fitting stress relaxation characteristics to Eq. 1 for different values of ${F}_{0}$. **d** *C*onstant *C* as a function of ${F}_{0}$. Dashed lines in (**c**) and (**d**) are linear fits. **e** Microscopy images of the contact area for four values of ${F}_{0}$. These images have been taken $t=60$ s after the squeezing was applied. Colored symbols indicate which data points in (**d**) correspond to which images in (**e**).

Our setup for stress relaxation experiments is schematically shown in Fig. 1a. A polymer sphere is held gently between a steel and a glass plate. The polymer materials used here are polypropylene and polytetrafluoroethylene which have existed in their metastable equilibrium state for a long time before the experiments. The top steel plate is attached to a rheometer to measure the pressing force. The rheometer has a large internal spring constant so that the gap does not change after the stress relaxation in polymer spheres. For the same reason, the bottom glass slide is attached to a strong custom-made frame. We decrease the gap (at a speed of roughly 1 mm/s) to squeeze the polymer sphere while the rheometer measures the normal force F and the microscope takes images (see “Methods”).

Two typical examples of the relaxation of the force that the sphere exerts on the plates as a function of time *t* are shown in Fig. 1b. Under a small load of 0.12 N, only surface plastic deformations are possible. On the other hand, under 53.05 N the bulk of the sphere deforms non-reversibly (Supplementary Fig. 1). However, in both cases, after a brief onset (*t* < 1 s) the force relaxation is logarithmic over many decades in time:

1 $$F(t)=B-C{{{{{\rm{ln}}}}}}\left(\frac{t}{1\,s}\right),$$

where *C* and *B* are constants. Previously, other molecular and/or athermal disordered systems including creased sheets^26,27^, crumpled papers^28,29^, and foams^29^ were also reported to display such logarithmic mechanical response to deformation. Moreover, enthalpy and specific volume measurements during the aging of polymers show a similar logarithmic behavior^30^. In the case of creased polymeric (Mylar and paper) sheets, which contain lines of localized plastic deformations, analysis of the relationship between *C* and *B* revealed that the logarithmic process is governed by a constant that doesn’t change with the sheet thickness or the applied load^27^. Similarly, for our polymer spheres, *B* is proportional to *C* for the whole range of the squeezing forces, from 0.1 N to above 50 N (Fig. 1c). As a result, we can write $B=C{{{{{\rm{ln}}}}}}\left(\frac{\tau }{1\,s}\right)$, where ${{{{{\rm{ln}}}}}}\left(\frac{\tau }{1\,s}\right)=22.17\pm 0.06$ is the slope of the plot ($\tau=135\pm 8$ years), irrespective of whether bulk or surface plastic deformations occur (the error corresponds to the uncertainty in the slope). Putting this equation into Eq. 1, $F$ can be expressed as $F=-C{{{{{\rm{ln}}}}}}\left(\frac{t}{\tau }\right)$. Then, in Fig. 1d, we plot *C* as a function of the initially applied (the maximum) force ${F}_{0}$, which again yields a simple linear proportionality, $C=n{F}_{0}$, so that:

2 $$F(t)={-{nF}}_{0}{{{{{\rm{ln}}}}}}\left(\frac{t}{\tau }\right),$$

where $n=0.0417\pm 0.0002$ (the error is the uncertainty in the slope). This expression is valid over the full range of forces, even though the corresponding contact areas are widely different (Fig. 1e and Supplementary Fig. 2). For example, the contact area corresponding to ${F}_{0}=$ 0.12 N consists of small islands determined by the surface roughness of the sphere while the contact image corresponding to ${F}_{0}=$ 53.05 N displays an almost complete Hertzian-type contact circle. It should be noted that the uncertainty in determining $F(t)$ close to the singularity point $t=0$ does not significantly change the obtained values for $\tau$ and $n$ (Supplementary Fig. 3).

**Fig. 2:**
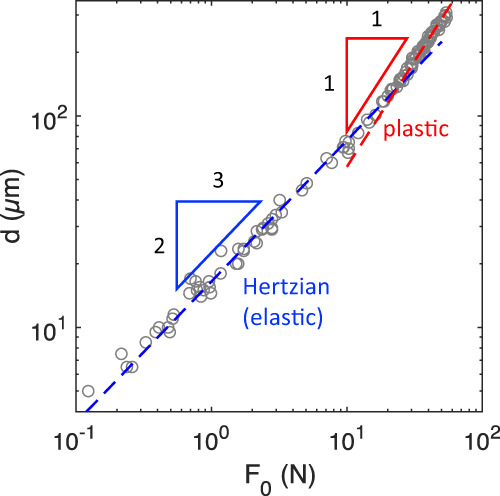
Failure of the bulk polymer. Deformation *d* of polypropylene spheres (as shown in Fig. 1a) as a function of the initially applied compressive force ${F}_{0}$. At small forces (${F}_{0} < 10{N}$), *d* increases with ${F}_{0}$ to the power of 2/3 (blue dashed line), corresponding to Hertz’s theory for elastic deformation. At larger forces, this changes into a linear increase (red dashed line), corresponding to plastic deformation (see Supplementary Fig. 10 for a linear-linear plot).

To determine when the transition from elastic to plastic deformations occurs in these experiments, we plot the deformation *d* of the sphere against the initial compressive force ${F}_{0}$ exerted on it (Fig. 2). For small forces, we find ${F}_{0}\propto {d}^{3/2}$ which corresponds to Hertz’s contact theory for bulk elastic deformation. For larger forces, the plot shows a transition to a linear behavior associated with plastic deformation. The intercept of fits to the elastic and plastic regimes can be considered as the point where the bulk of the sphere yields to flow^10^.

Next, we focus on the regime of small compressive forces, where plastic deformations are limited to the sphere’s surface asperities (Supplementary Fig. 8). The surprise is that any configuration of the real contact area, which is determined by the details of the surface irregularities, behaves according to Eq. 2. This signifies that Eq. 2 remains valid locally. Consequently, in a frictional contact that involves a range of microscopic contact forces^31^, all the forces acting within the interface will dissipate at the same rate.

### Frictional aging

**Fig. 3:**
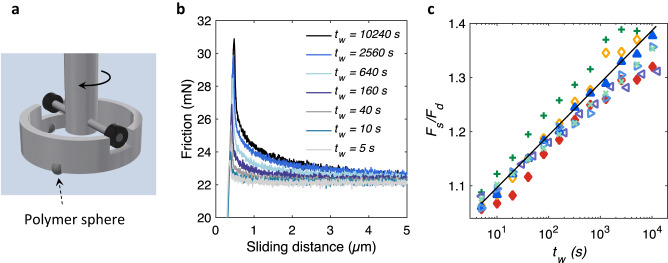
The interface aging and the evolution of static friction. **a** Schematic of the apparatus for the friction measurements. A rheometer is used to rotate a hollow tube around its symmetry axis. Three polymer spheres are attached to the underside of the tube at equal distances, and these, together with a substrate at the bottom, form the frictional interfaces. **b** Friction as a function of sliding distance for polypropylene spheres on glass under the combined weight of the tube and spheres (42 mN) after different waiting times *t*_*w*_. The polymer spheres are not replaced between these measurements. **c** The ratio of static friction (*F*_*s*_, the peak friction value) to dynamic friction (*F*_*d*_, mean friction in steady-state) for multiple aging experiments as in (**b**): solid triangles are from curves in (**b**), open right triangles correspond to a subsequent repeat of the experiment with the same spheres, open left triangles are with new polypropylene spheres, $\times$ signs are with a new tube of 87 mN weight, and red diamonds correspond to an experiment on a silicon wafer. In all the above experiments, the imposed sliding velocity is 86 nm/s. + signs and open diamonds are repeats of the last two experiments, respectively, with a sliding velocity of 258 nm/s. The solid line corresponds to equation ${F}_{s}\left(t\right)={F}_{\tau }+n{F}_{{{{{{\rm{d}}}}}}}{{{{{\rm{ln}}}}}}\left(\frac{{t}_{w}}{\tau }\right)$ with $n=0.0417$ determined from the stress relaxation experiments (ln is natural logarithm). The free parameter ${F}_{\tau }$ is the static friction at time $t=\tau$. Here, $\frac{{F}_{s}}{{F}_{d}}=1+0.0417\,{{{{{\rm{ln}}}}}}\,\left(\frac{{t}_{w}}{1{{{{{\rm{s}}}}}}}\right)$.

We now investigate whether the frictional strength, as a collective macroscopic observable of the evolution of a static interface, also evolves at the same rate as the relaxation of the local microscopic normal stresses. Our apparatus for friction measurements is shown in Fig. 3a. We use a rheometer with a custom-made tool to rotate a cylindrical tube with three polymer spheres glued to its underside, while it is measuring the applied torque. Figure 3b shows typical examples of friction as a function of sliding distance for polypropylene spheres on a glass substrate with different waiting times *t*_*w*_ before the measurement. As generally expected, the static or peak friction *F*_*s*_ grows with waiting time *t*_*w*_. Figure 3c plots *F*_*s*_ normalized by the dynamic or steady-state friction *F*_*d*_ as a function of aging time *t*_*w*_ for multiple experiments. These data are in close agreement with the stress relaxation rate $n=0.0417$ obtained from squeezing experiments (solid line), indicating that frictional aging is given by stress relaxation.

**Fig. 4:**
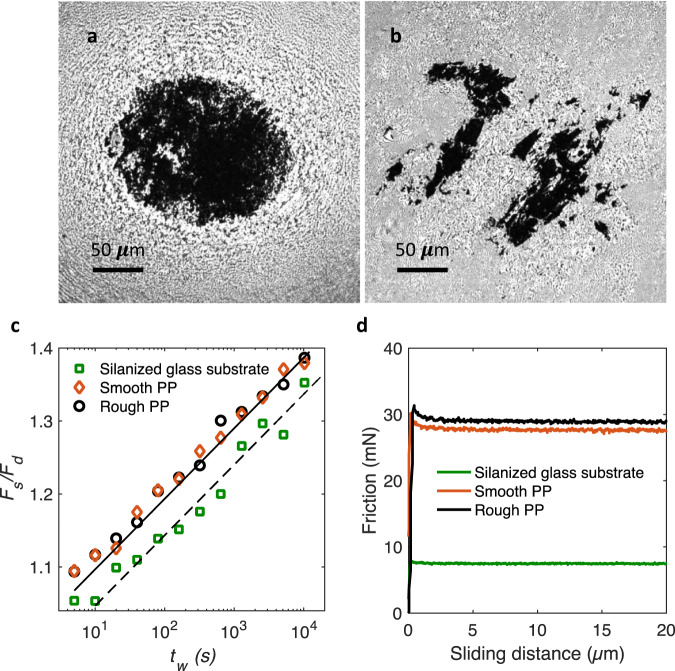
Large changes in surface topography and chemistry do not affect the frictional aging rate. **a** Smooth and **b** rough polypropylene spheres (Supplementary Fig. 4) with root-mean-square (rms) roughness heights of 0.23 and 1.81 μm, respectively, have very different real contact areas under the same normal force of 0.35 N. **c** These polypropylene (PP) spheres, however, show the same aging characteristics as for the pristine spheres with rms-roughness of around 0.63. The roughness values are obtained after correcting for the macroscopic spherical curvature. The solid line is, as in Fig. 3c, $\frac{{F}_{s}}{{F}_{d}}=1+0.0417\,{{{{{\rm{ln}}}}}}\,(\frac{{t}_{w}}{1{{{{{\rm{s}}}}}}})$. The frictional aging for the polypropylene spheres on a silanized glass substrate (green squares) is also consistent with the slope ${{n}}=0.0417$ determined from the bulk stress relaxation experiments, although in this case, there is a clear vertical shift associated to decrease of the friction due to the change in surface chemistry. The dashed line is $\frac{{F}_{s}}{{F}_{d}}=0.95+0.0417\,{{{{{\rm{ln}}}}}}\,(\frac{{t}_{w}}{1{{{{{\rm{s}}}}}}})$. **d** While silanization of the glass substrate does not change the frictional aging rate, it reduces the absolute dynamic friction by a factor of 3 to 4.

The observed frictional aging rate is independent of the details of the surface roughness, the imposed velocity (typically <1 µm s^−1^), and the underlying surface interactions; showing unambiguously that the frictional aging is due to the bulk stress relaxation of the polymer. For frictional aging of the polypropylene spheres on an atomically smooth silicon substrate, the same value of $n \, \approx \,0.042$ is found again (Fig. 3c). Figure 3c also shows that increasing the speed by a factor of three also does not change the measured frictional aging rate on either of glass or silicon substrates. Furthermore, in order to check that the frictional aging rate does not depend on the real area of contact, we changed the surface roughness of the polymer spheres (Supplementary Fig. 4). Polypropylene spheres with very different surface topographies and hence very different contact areas for the same force (Fig. 4a, b) still yield the same frictional aging (Fig. 4c). This means that frictional aging is not sensitive to the details of the contact area, in line with our stress relaxation measurements (Fig. 1). In addition, we perform a control experiment changing the surface chemistry by silanizing the glass substrate, which increases the water contact angle of the glass substrates from below 10° to above 150°. We find that although the change in surface chemistry reduces the measured friction by a factor of 3 to 4 (Fig. 4d), no change in the frictional aging rate is observed again: the slope of the dashed line in Fig. 4 is identical to that of the bare substrate. This unequivocally confirms that frictional aging is a bulk polymer property. Interestingly, other studies on soft materials obtained similar values for the aging rate; $n \, \approx \, 0.040$ for Bristol board^32^. For hard materials, on the other hand, smaller values are found; $n \, \approx \, 0.012$ for quartz sandstone^33^ [$n=\frac{b}{a}$ in the equation ${F}_{s}=a+b\,{{{{{\rm{ln}}}}}}(\frac{t}{1{s}})$].

## Discussion

Our results for polypropylene spheres (and repeated for polytetrafluoroethylene spheres, see Supplementary Figs. 5–7) not only demonstrate that the aging of frictional interfaces of polymers originates from the stress relaxation of the bulk material, but also provide a quantitative description of these relaxation dynamics. We obtain a stress relaxation constant from bulk relaxation measurements that directly predicts the evolution of the static friction in time. The observation that the microscopic compressive forces within plastically deformed areas in contact interfaces undergo a generic material-dependent relaxation could also provide a basis for a theoretical understanding of the time dependence of macroscopic adhesion between solids^5,7,34^. Especially, the adhesion hysteresis which has already been qualitatively attributed to viscoelastic dissipation might be addressed through our results.

## Methods

### Stress relaxation measurements

Our experiments were conducted using polypropylene spheres of diameter 2.45 mm (www.cospheric.com) or polytetrafluoroethylene (PTFE) spheres of diameter 3.18 mm (www.goodfellow.com). For stress relaxation experiments (Fig. 1a), the polymer sphere is initially fixed on a glass slide by a small holder from the side, so that we can align it with an underneath inverted microscope. Then the upper steel plate is lowered in micrometer steps to touch the sphere with a small force (<2 mN). Afterward, the holder is removed and the sphere is free laterally in case of expansion under the squeezing force. The top steel plate is connected to a rheometer (Anton Paar, DSR 502) that for this experiment is used only as a dynamometer. The advantage of the rheometer for our force measurement is that it has a large internal spring constant by design. Therefore, the changes in the working gap during the relaxation process which will occur with the transfer of the elastic energy stored in the rheometer to the sphere is negligible. Moreover, the rheometer itself does not show any internal relaxation (checked by a fully elastic stainless-steel spring). The bottom glass slide is also on top of a stiff custom-made steel frame instead of a usual microscopy stage. We lower the upper plate and rheometer head by a value of *d* measured by a displacement sensor (ID-H0530 Digimatic Indicator, Mitutoyo) with a speed of roughly 1 mm/s. The plates press the polymer sphere while the rheometer is measuring the normal force with a rate of 0.1 s and the microscope (ZEISS Axiovert 200M LSM 5 Pascal) is taking images (one frame per second).

### Friction experiments

A cut of an aluminum cylindrical tube (outer diameter of 30 mm, wall thickness of 2.5 mm) with three polymer spheres glued equidistantly to its underside lays on a glass (or silicon) wafer (Fig. 3a). The tube has two slots on the upper side which is used to rotate it by a custom-made rheometer tool connected to an Anton Paar MCR 302 rheometer. The connection between the tube and the rheometer tool is through frictionless bearings so that the normal load on the polymer-glass frictional interface is given only by the weight of the tube and spheres. This indirect connection which allows the tube to adjust freely in the normal direction is important. Because, otherwise, any small misalignment between the tube and the bottom glass substrate could cause a large change in the normal stresses within the frictional interfaces. After a certain waiting/aging time t_w_, the rheometer rotates the tool and drags the spheres on a circular path on the glass while measuring the rotational displacement and the applied torque (minimum 10 nNm) simultaneously. The rheometer can achieve angular velocities down to 10^−9 ^rad s^−1^ depending on the sampling time (Anton Paar), which is much below the rotational speeds needed in this work (10^−6 ^s^−1^). The moment arm of the frictional force is 13.75 mm, which is much larger than a typical Hertz contact circle (<100 ${{{{{\rm{\mu }}}}}}{{{{{\rm{m}}}}}}$ in diameter in normal forces less than 100 mN) and the sliding distance in our experiments.

### Modifying the surface topography of polypropylene spheres

Smooth polypropylene surfaces were obtained by polishing the underside of the spheres glued to the tube by using lens paper. Rough surfaces were produced by pressing them (normal force <200 mN) multiple times and randomly against a rough sandblasted glass substrate. Supplementary Fig. 4 shows typical surface images of these spheres taken using a 3D laser scanning microscope (Keyence VK-X1000).

### Silanization of glass substrates

To have superhydrophobic glass substrates (water contact angle > 150°), they were treated with a 1% solution of trichloro(octyl)silane in toluene. After cleaning and plasma treatment, the glass substrates were immersed in the solution for 30 min, followed by rinsing with isopropanol and drying in a stream of nitrogen gas.

## Supplementary information

Supplementary Information

Peer Review File

## Data Availability

The data that support the findings of this study are available in the article and its Supplementary Information. Source Data has been deposited in figshare under the accession code (10.6084/m9.figshare.23096219).
