# Supplementary material for: Predicting frictional aging from bulk relaxation measurements
Source: Nat Commun. 2023 Jun 17;14:3606. doi: 10.1038/s41467-023-39350-3 (PMC10276840; doi:10.1038/s41467-023-39350-3)
Supplement: Supplementary file 1 — Supplementary Information [file 41467_2023_39350_MOESM1_ESM.pdf]

Supplementary Information for:
Predicting frictional ageing from bulk relaxation measurements

Kasra Farain[1] and Daniel Bonn[1]*

[1]Van der Waals–Zeeman Institute, Institute of Physics, University of Amsterdam; Science Park 904, 1098 XH Amsterdam, Netherlands.

*Corresponding author. Email: d.bonn@uva.nl

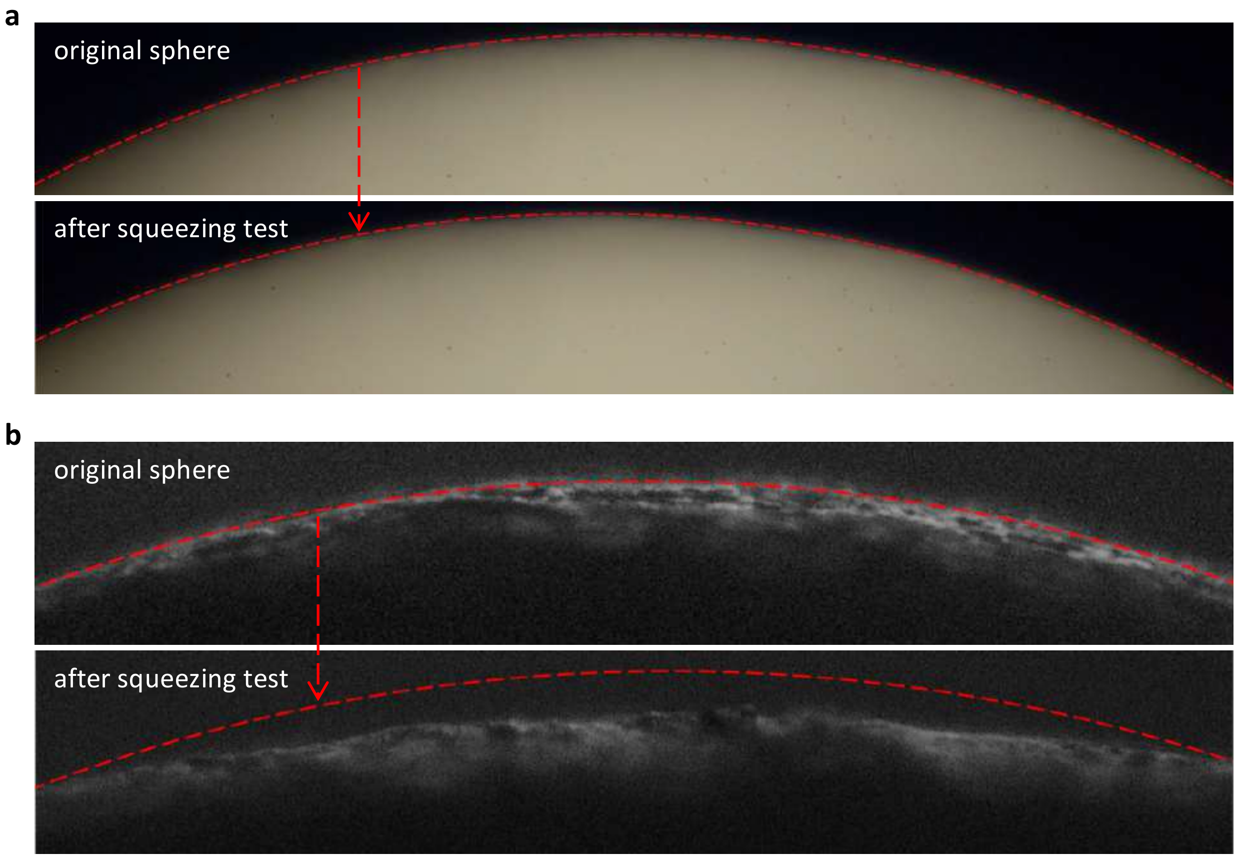

Supplementary Fig. 1| Bulk deformation of the polymer spheres. a, A polypropylene sphere before and after a squeezing test in which the compressive force $F_0 = 4.31$ N does not satisfy the bulk yield criteria. **b,** Change of the radius of curvature for a typical PTFE sphere after a squeezing test in which the compressive force $F_0 = 19.23$ N exceeds the bulk yield criteria (see Supplementary Fig. 6).

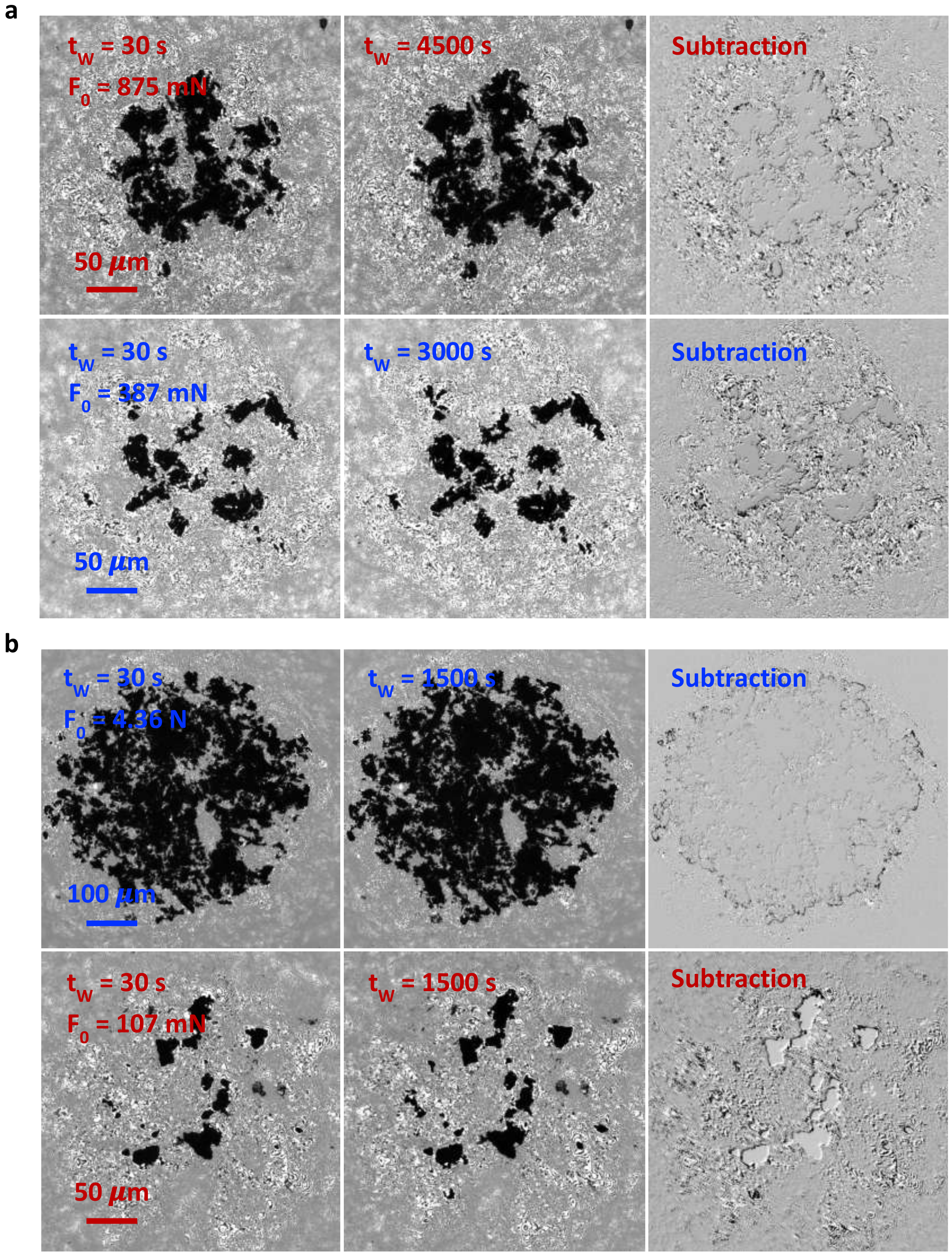

Supplementary Fig. 2| Contact area changes in time. Typical contact area images of **a,** polypropylene and **b,** PTFE spheres on glass substrate. The normal load in each case is determined on the left panel. t_W is the time interval between establishing the contact and taking the image. The right panels are the subtraction (using ImageJ) of the two left images taken at different times which show their difference is very small and difficult to quantify.

Time (s)	Force (N)
39.2	-0.000349
39.3	-0.00052
39.4	0.000401
39.5	-0.000359
39.6	-0.000681
39.7	-0.000616
39.8	-0.000659
39.9	2.766138
40	25.895792
40.1	30.370847
40.2	29.548187
40.3	29.108877
40.4	28.773466
40.5	28.533077
40.6	28.359706
40.7	28.211832
40.8	28.057592

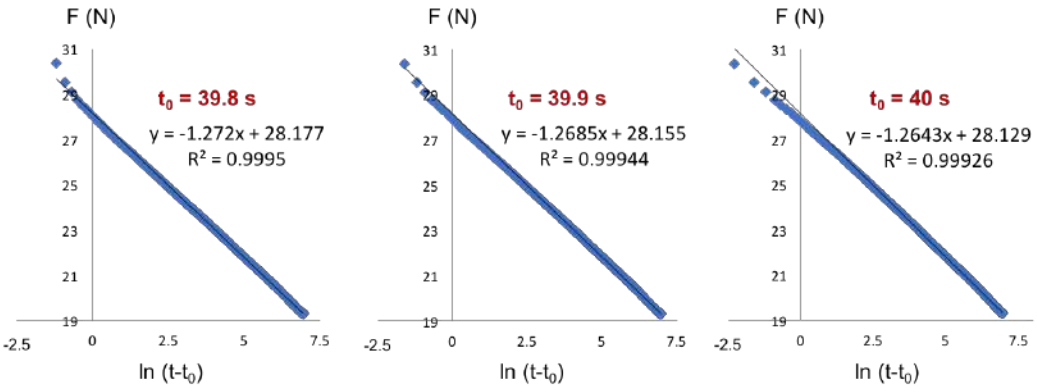

Supplementary Fig. 3| Uncertainty in t_0 does not significantly change the constants C and B. The result of a typical stress relaxation test is fitted to $F(t) = B - C\ln(t - t_0)$ with three different t_0, the time at which the squeezing is applied (data points around t_0 are shown in the table). $t = 0$ is the time at which the rheometer has started the measurement. $B \approx 28$ N and $C \approx 1.27$ N from all these fits.

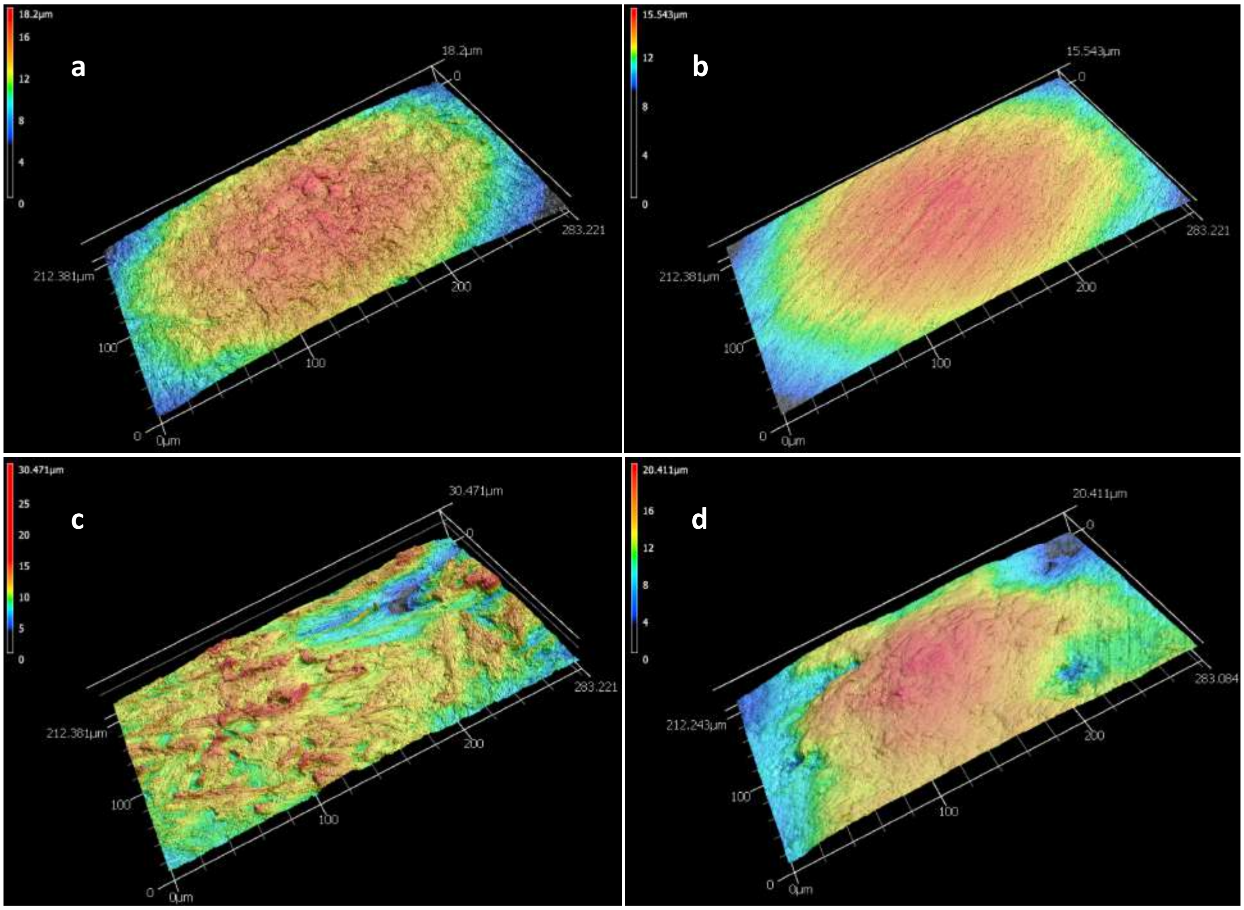

Supplementary Fig. 4| Surface roughness images of the polymer spheres. Surface topography of **a**, pristine **b**, smooth and **c**, rough polypropylene spheres. Root-mean-square (rms) roughness heights, obtained after removing the macroscopic spherical curvatures, are 0.63, 0.23, and 1.81 µm, respectively. **d**, Surface topography of a typical PTFE sphere (pristine). Root-mean-square roughness height in this case is 1.89 µm. These images were taken using a 3D laser scanning microscope (Keyence VK-X1000), and the rms-roughness values are calculated using the Keyence viewer application.

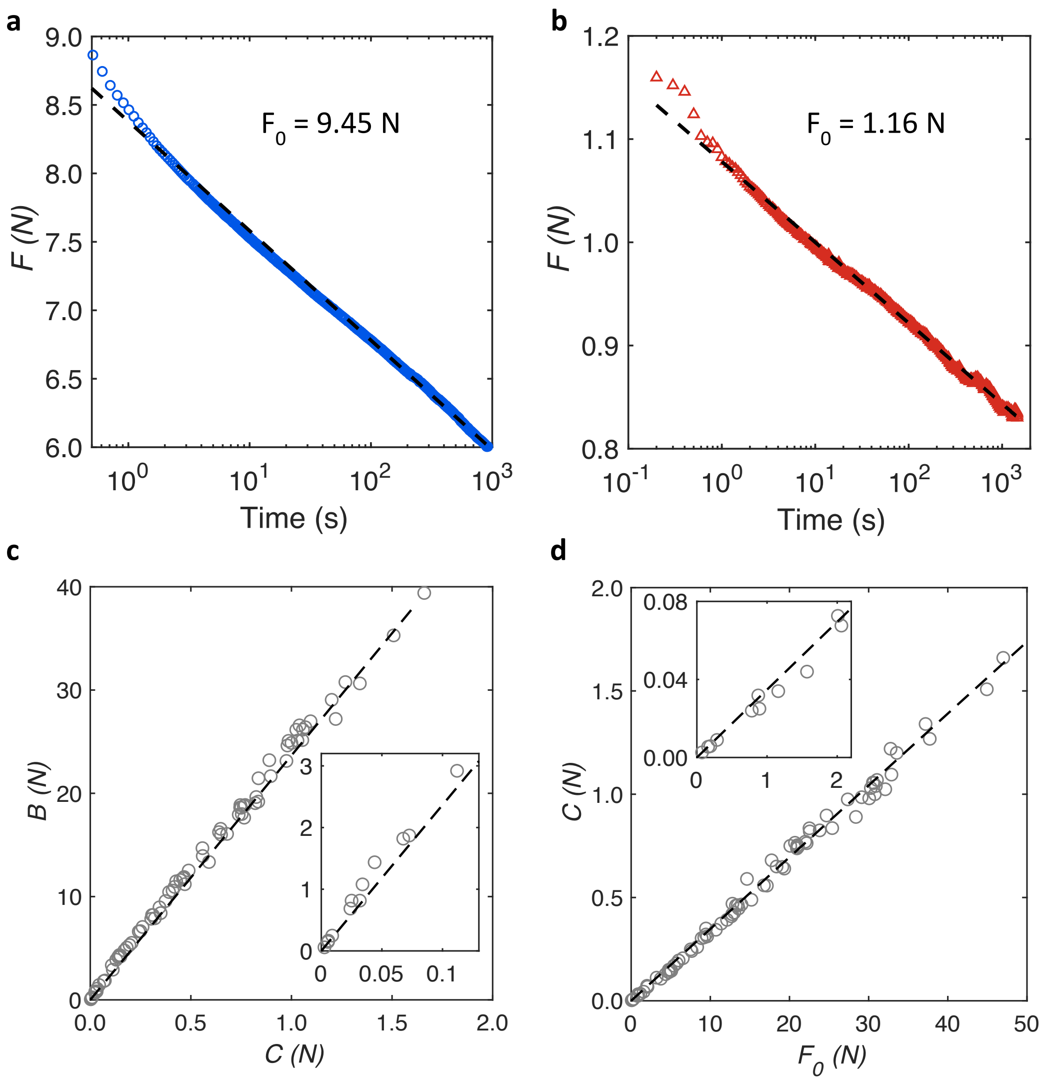

Supplementary Fig. 5| Stress relaxation of PTFE spheres after bulk and/or surface deformations. a and **b**, Relaxation of the compressive force F applied to a PTFE sphere at constant strain for two typical examples with the initial forces of 9.45 N and 1.16 N. **c**, Relation between constants B and C as obtained from fitting stress relaxation characteristics of PTFE spheres to equation $F(t) = B - C\ln(\frac{t}{1\,s})$. Dashed line is linear fit with the slope of $\ln\left(\frac{\tau}{1\,s}\right) = 24.39 \pm 0.12$. Therefore, $\tau = 1241 \pm 149$ years for PTFE spheres. **d**, The constant C as a function of the initially applied normal force F_0, which again yields a simple linear proportionality, $C = nF_0$. The linear fit (dashed line) yields $n = 0.0344 \pm 0.0002$.

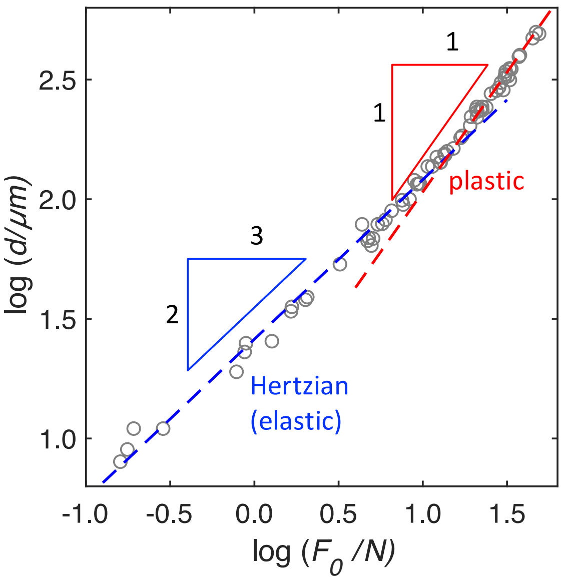

Supplementary Fig. 6| Bulk deformation of PTFE spheres. Deformation d of a PTFE sphere subjected to an initial compressive force F_0 is shown. The blue dashed line corresponds to Hertz equation for elastic deformation. The red dashed line, at higher forces, associates to a plastic behavior.

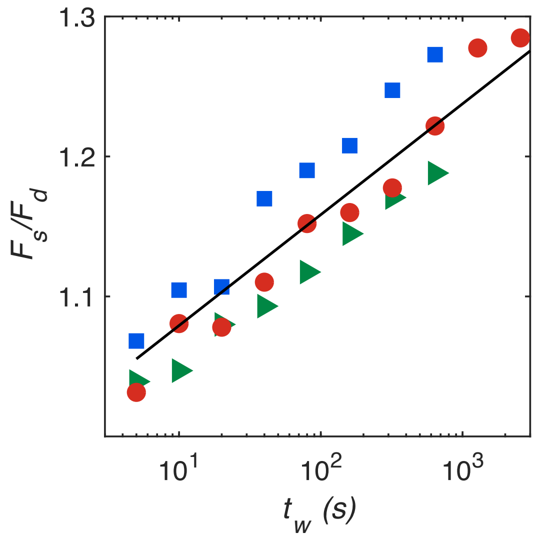

Supplementary Fig. 7| Frictional aging of PTFE spheres. The ratio of static friction (F_s, the peak friction value) to dynamic friction (F_d, mean friction in steady-state) for three typical sliding experiments with PTFE spheres on glass substrate under 28.4 mN normal load as a function of the stationary aging time t_w before the sliding. The imposed sliding velocity is 86 nm/s. The solid line corresponds to equation $F_s(t) = F_\tau + nF_d \ln\left(\frac{t}{\tau}\right)$ with $n = 0.0344$ determined from the stress relaxation experiments (see Supplementary Fig. 5). The free parameter F_τ is the static friction at time $t = \tau$. Here, $\frac{F_s}{F_d} = 1 + 0.0344 \ln\left(\frac{t}{1\ s}\right)$

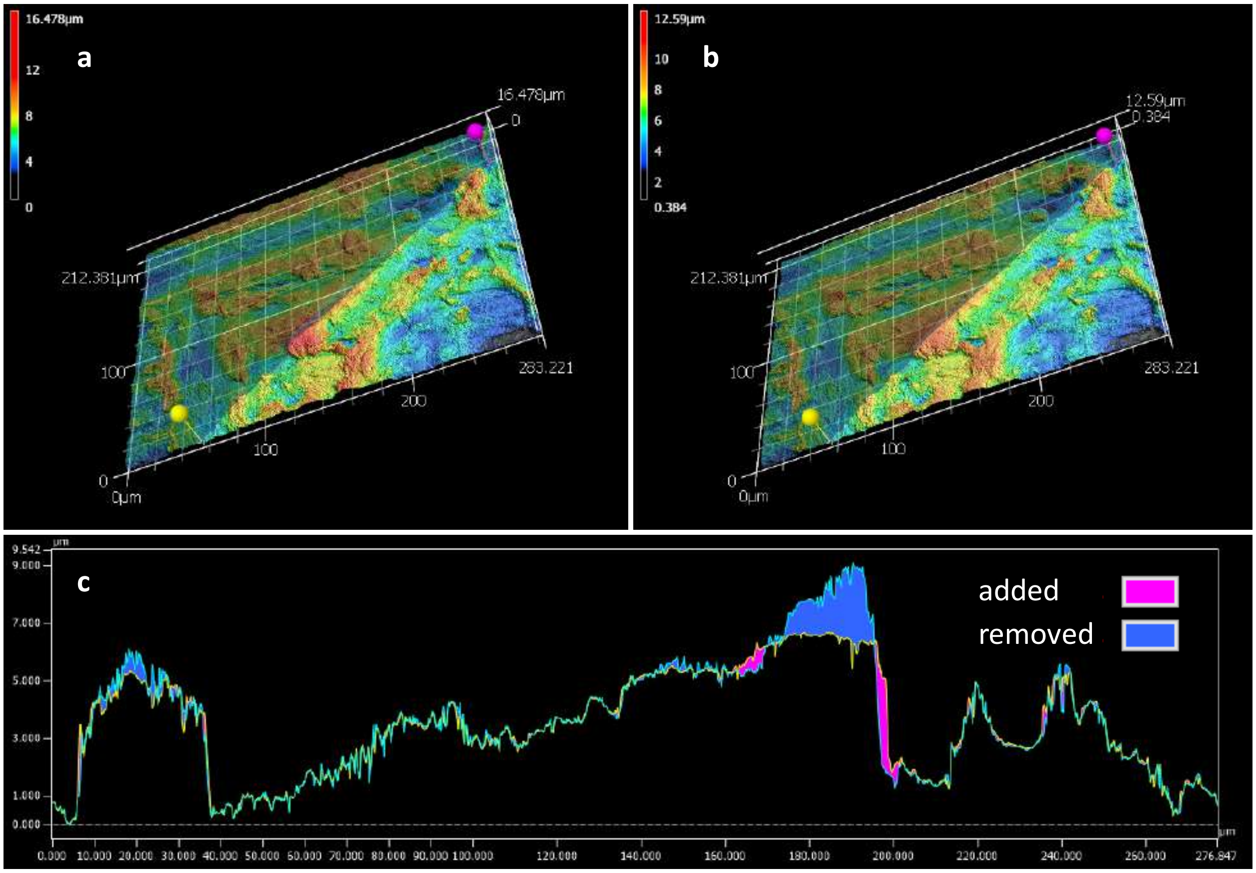

Supplementary Fig. 8| Surface plastic deformations. Rough polypropylene surface **a**, before and **b**, after pressing against a glass substrate under an initial normal load of 380 mN for 10 min. **c**, The changes of surface roughness on the line specified in **a** and **b**.

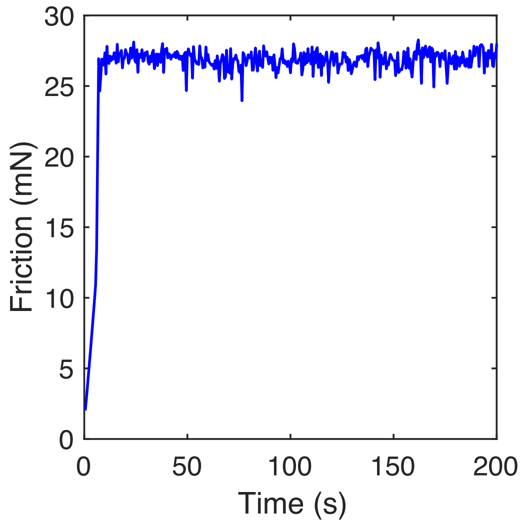

Supplementary Fig. 9| Steel spheres do not show frictional aging on silicon substrate. Sliding friction of steel ball bearings (in the same setup as Fig. 3**a**) on atomically smooth silicon substrate after 30 min aging. Steel does not show stress relaxation, and consequently does not have frictional aging. The sliding speed in this experiment is 86 nm/s.

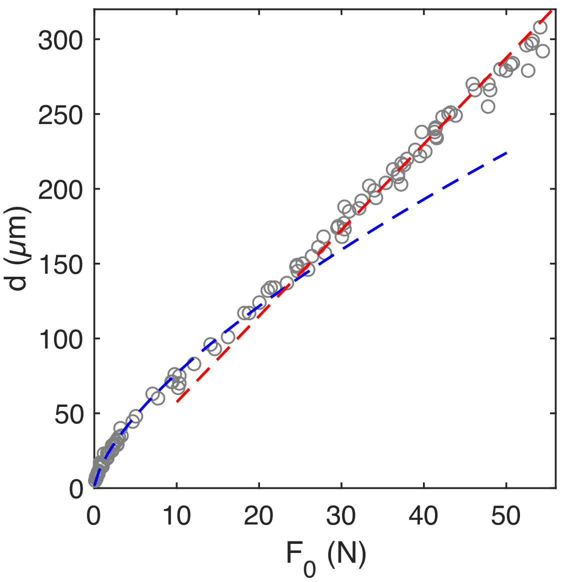

Supplementary Fig. 10| A linear-linear plot of Fig. 2. Deformation d of polypropylene spheres as a function of the initially applied compressive force F_0.
